# 

**Published:** 1893-07

**Authors:** 


### CONTENTS FOR JULY, 1893.

PAGE.

ORIGINAL ADDRESS.

Children's Rights. By I. N. Love, M. D. .......... 709

ORIGINAL COMMUNICATIONS.

Some of the Causes of Indigestion and Malnutrition in Nursing Infants. By Irving M. Snow, M. D. .......... 726

The Careless Examination of Urine. By Frederick H. Stanbro, M. D. .......... 736

SOCIETY PROCEEDINGS.

American Association of Obstetricians and Gynecologists .......... 740

CLINICAL REPORT.

A Cyst of the Cerebellum .......... 748

ABSTRACT.

Neuritis and Myelitis and the Forms of Paralysis and Pseudo-Paralysis Following Labor .......... 750

EDITORIAL.

The Relation of Noise to Good Health .......... 754

Topics of the Month .......... 755

Personal .......... 760

Society Meetings .......... 760

Book Reviews .......... 761

### Index to Advertisements.

PAGE.

PAGE.

